# Retraction: Exploration of multiple signaling pathways through which sodium tanshinone IIA sulfonate attenuates pathologic remodeling experimental infarction

**DOI:** 10.3389/fphar.2025.1575629

**Published:** 2025-02-19

**Authors:** Frontiers Editorial Office

The journal retracts the 12 July 2019 article cited above.

Following publication, concerns were raised regarding the integrity of the images in the published figures. Image duplication was identified between Figure 6B and 7B. Some panels of Figure 3C, 6B and 9B were also found to be published in other articles.

The authors failed to provide a satisfactory explanation during the investigation, which was conducted in accordance with Frontiers’ policies. As a result, the data and conclusions of the article have been deemed unreliable, and the article has been retracted.

This retraction was approved by the Chief Editors of Frontiers in Pharmacology and the Chief Executive Editor of Frontiers. Author MV agrees to this retraction. Authors SM, MC, MZ and AH did not respond to correspondence regarding this retraction.

Frontiers would like to thank the users on PubPeer for bringing the published article to our attention.

